# Endophytes: Improving Plant Performance

**DOI:** 10.3390/microorganisms10091777

**Published:** 2022-09-03

**Authors:** Camilla Fagorzi, Alessio Mengoni

**Affiliations:** Department of Biology, University of Florence, Via Madonna del Piano 6, 50019 Sesto Fiorentino, Italy

Endophytes represent microorganisms that reside within plant tissues, without typically causing adverse effects to the plants, for a substantial part of their life cycle, and are primarily known for their beneficial role to their host plant [1]. Specific endophytes may have evolved with their host as a holobiont, providing excellent working models for evolutionary theory or could be a source of yet unknown novel molecules, with potential pharmaceutical, agricultural or environmental significance [2,3,4].

The discovery of endophytes and the recognition of their relevant role dates to 1866, when De Bary detected microbial cells inside plant tissues [5]. The first microorganisms to be termed endophytes were fungi; only later were bacteria found to be massively present in plant tissues [6]. Extensive studies defined subgroups of endophytic communities, with ‘obligate’ or ‘facultative’ microorganisms, depending on the metabolites of the plant for survival in the first case, or being able to associate with the plant from the rhizosphere and atmosphere in the second one [7,8,9]. Estimates of microbial community density range around orders of magnitude of 10^3^–10^4^ bacterial cells per gram of tissue, but in some parts (e.g., root nodules) it can also reach an order of magnitude of 10^7^ [10,11,12].

A bibliographic search with the keyword “Endophyte” on the PubMed database from 1946 to 2022 sorted out a total of 12,372 entries, while 9547 and 3410 entries are sorted for more specific “Fungal Endophytes” and “Bacterial Endophyte”, respectively (16 August 2022). While for “Endophytes” (manly fungi) already in 1946 two papers are indexed, the first paper indexed with “Bacterial Endophytes” dates to 1975. Indeed, only later it was recognized that many bacterial taxa colonize the internal tissues of plants, without harming them. In the last 15 years, an increasing trend of research papers is witnessing the interest of the scientific community toward microbial endophytes (Figure 1), mainly for fungal endophytes, though interest in bacterial endophytes is increasing.

Endophytes are studied for different purposes, spanning from the search for relevant activities (e.g., biocontrol, bioremediation, metabolite production, plant growth and resistance) to ecological investigation, including adaptation to harsh conditions. For instance in medicinal plants, endophytes have been shown to participate in or modulate the biosynthesis of bioactive molecules [13,14]. In heavy-metal accumulating plants, bacterial endophytes have evolved resistance to the toxic effects of metals [15], while in plants that contain antimicrobial metabolites (e.g., many aromatic plants) the endophytic microbiome shows specific adaptation toward the essential oil of the different plant chemotypes [16].

Where do endophytes come from? Clearly, for many endophytes, the soil is their original habitat. Once the presence of the plant roots is perceived, many bacteria are attracted and proliferate around the roots, also forming biofilms over the root surface. These new habitats are called rhizosphere and rhizoplane, meaning the soil environments close to the roots and the root surface, respectively [17].

From there, they may eventually penetrate the root tissue in presence of cracking of the root epidermis [18], but some of them have developed specific signaling to enter the plant and become symbionts (e.g., rhizobia). However, microbes are present in the dust particles also and can be brought by animals (e.g., pollinating insects). Consequently, endophytes may originate from the phyllospheric microbiome (the leaf’s surface microbiome) [19] or from the floral microbiome [20]. Endophytes originating from the floral microbiome are particularly intriguing since they can in theory propagate via seeds, then showing vertical transmission with their host plant. Indeed, seed endophytes are gaining the attention of several investigators since they could be nice examples of coevolution between host and microbe, but also a powerful way to produce seeds of crops that may harbor plant-growth-promoting microbes [21]. Such applications need detailed knowledge of the peculiarities of a microbial endophyte. In fact, not all rhizosphere or phyllosphere microbes can be endophytes. Adaptation to plant tissues seems to require a specific genetic repertoire, including metabolic and signaling functions [22,23]. However, a large part of the interest toward endophytes is focused on the ability of many of them to alleviate plant abiotic stress [24], promote plant growth and antagonize pathogens (biocontrol) [1]. The isolation and characterization of large culture collections of endophytes is constituting an important resource for sustainable food production and low-input agriculture, facing the need of exploiting marginal lands and increasing the yield of crops in a neutral climate change way [25]. However, a lot of work has still to be done, since the number of questions requires proper answers prior to a rational application of microbial endophytes in improving plant performances [26,27,28]. Among the outstanding questions to be answered, priorities can be given to the identification of a common set of microbial endophytes to be used as bioinoculants, to the development of setups and methods to translate knowledge from model to nonmodel plants (hence from lab-to-the-field) and to the elucidation of possible “rules of engagement” (genetic and physiological) of being an endophyte. With this Special Issue, we aim to stimulate research, opinions and reviews which, by addressing microbial endophytes, can promote the translation from laboratory discovery to field applications.

## Figures and Tables

**Figure 1:**
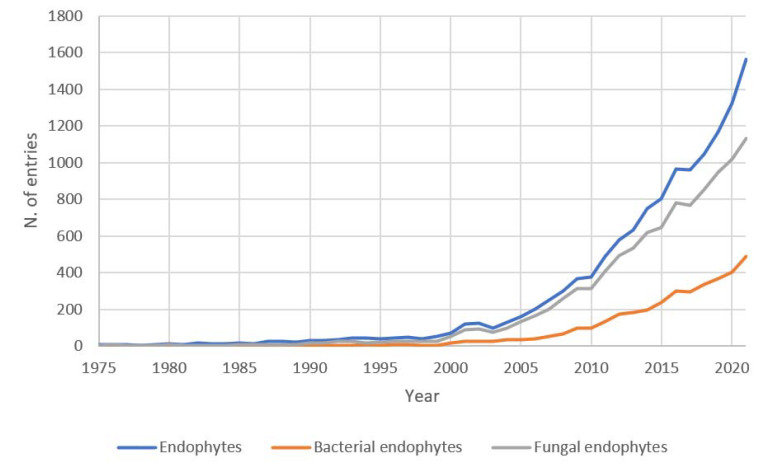
Number of entries by year (starting from 1975) present in PubMed database. Searches with keyword “Endophytes”, “Fungal Endophytes” and “Bacterial Endophytes” are shown.
